# 

**Published:** 1804-09-01

**Authors:** 


### To CORRESPONDENTS.

Communications are received from Dr. Uwins, Medicinæ Studiosus, and Mr. Thackeray.

